# Paraneoplastic Neuromyelitis Optica Spectrum Disorder Associated With Ascending Colon Cancer Expressing Aquaporin-4 and Glucose-Regulated Protein 78

**DOI:** 10.7759/cureus.104062

**Published:** 2026-02-22

**Authors:** Ryo Iwamoto, Ryoji Nakada, Ryota Sato, Shinji Miyagawa, Fumitaka Shimizu

**Affiliations:** 1 Department of Neurology, Jikei University Kashiwa Hospital, Chiba, JPN; 2 Department of Neurology and Clinical Neuroscience, Yamaguchi University Graduate School of Medicine, Ube, JPN

**Keywords:** aquaporin-4, colon cancer, glucose-regulated protein 78, neuromyelitis optica spectrum disorder, paraneoplastic syndrome

## Abstract

Neuromyelitis optica spectrum disorder (NMOSD) is a central nervous system demyelinating disease, typically defined by the presence of optic neuritis and longitudinally extensive transverse myelitis. A 69-year-old woman presented with acute longitudinally extensive transverse myelitis and motor and sensory deficits. Laboratory testing confirmed the presence of aquaporin-4 (AQP4) antibodies, leading to a diagnosis of NMOSD. Her symptoms improved following methylprednisolone pulse therapy. Further evaluation revealed concurrent ascending colon cancer. Surgical resection of the tumor was performed while the patient was maintained on 10 mg of prednisolone. Immunohistochemistry revealed high expression of AQP4 and glucose-regulated protein 78 (GRP78) in tumor cells. GRP78 antibodies were also detected in the serum. Postoperatively, both AQP4 and GRP78 antibodies became undetectable, and no recurrence of NMOSD was observed during a six-month follow-up while continuing prednisolone therapy. NMOSD results from astrocytic injury caused by AQP4 antibodies, which require disruption of the blood-brain barrier (BBB) to penetrate the nervous system. Recent studies have shown that GRP78 antibodies may contribute to BBB disruption. We hypothesize that an immune response against tumor-expressed AQP4 and GRP78 triggered the production of corresponding antibodies, with GRP78 antibodies contributing to BBB breakdown and the onset of paraneoplastic NMOSD.

## Introduction

Neuromyelitis optica spectrum disorder (NMOSD) is a severe, relapsing autoimmune inflammatory demyelinating disease of the central nervous system, typically characterized by optic neuritis and longitudinally extensive transverse myelitis. Aquaporin-4 (AQP4) autoantibodies are disease-specific markers for NMOSD, and their presence is required for diagnosis [1]. Relapse remains a significant clinical challenge. Preventive treatment typically includes corticosteroids, immunosuppressants, and biologics; however, these therapies do not entirely prevent relapse and are associated with notable side effects, including infection risk [2]. NMOSD develops when circulating AQP4 antibodies cross the blood-brain barrier (BBB) and cause astrocyte damage. Glucose-regulated protein 78 (GRP78) is a stress-inducible molecular chaperone that is upregulated under endoplasmic reticulum stress conditions and supports cellular survival by suppressing apoptosis. In addition to its intracellular role, GRP78 can be expressed on the cell surface, where it activates signaling pathways such as PI3K/AKT and NF-κB, thereby promoting cell proliferation and viability [3]. Shimizu et al. recently reported the presence of GRP78 antibodies in the sera of patients with NMOSD, suggesting a potential role in BBB disruption [3,4]. GRP78 is known to be expressed at higher levels in tumor cells than in normal cells [5,6] and is involved in tumor development and progression [7]. We report a case of NMOSD associated with ascending colon cancer expressing both AQP4 and GRP78, with positivity for both AQP4 and GRP78 antibodies, highlighting a possible paraneoplastic mechanism linking tumor-associated GRP78 expression with BBB disruption.

## Case presentation

**Figure 1:**
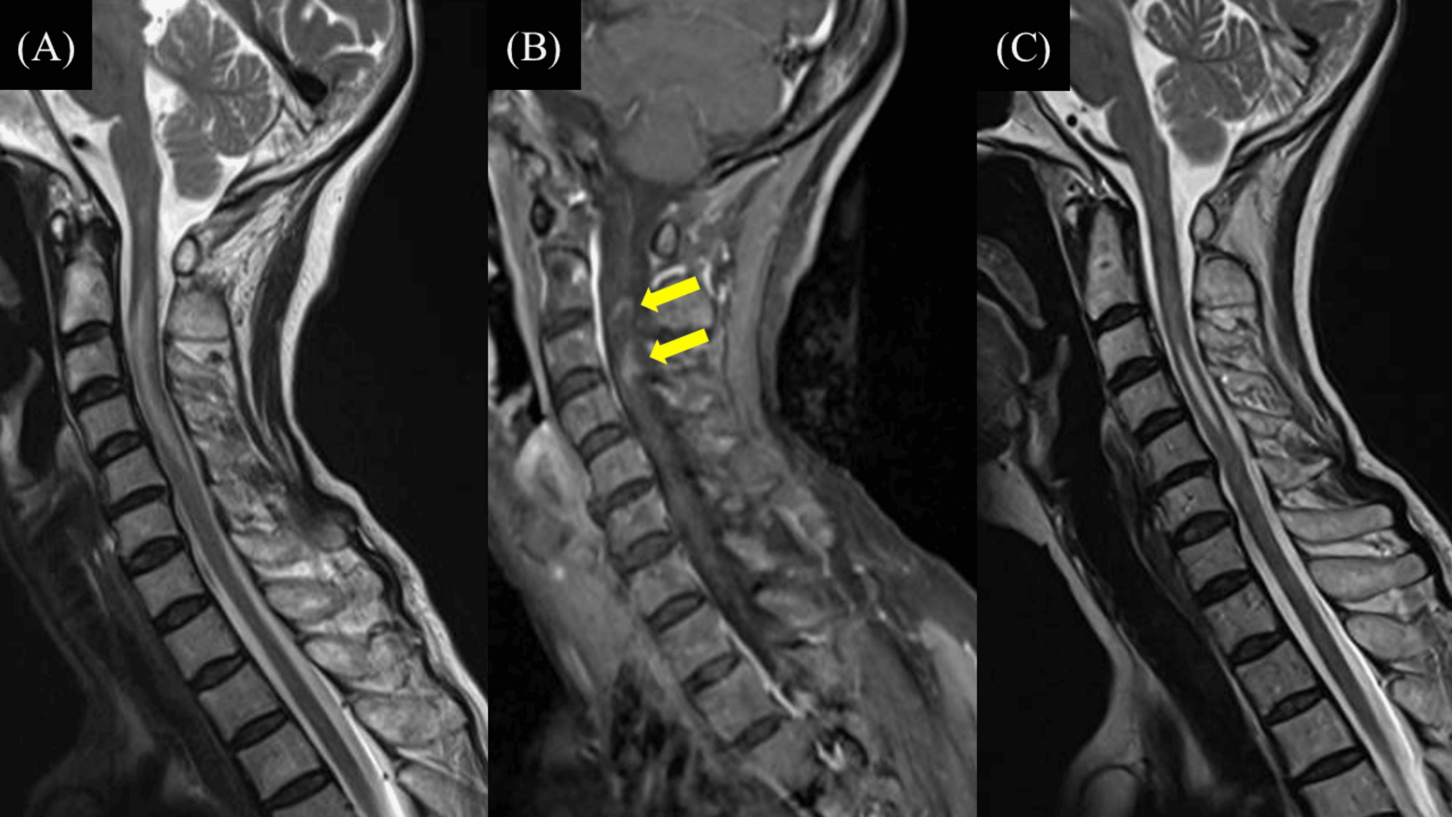
MRI revealing longitudinal spinal cord lesion from C2 to C5 and focal contrast enhancement at C3, consistent with neuromyelitis optica spectrum disorder. (A) T2-weighted images show hyperintense areas extending from C2 to C5 on sagittal images, and bright spotty lesions were detected on axial images. (B) Gadolinium-enhanced T1-weighted images demonstrate focal enhancement localized at the C3 level (arrows). (C) Post-treatment MRI shows a reduction in T2 hyperintense lesions in extent.

A 69-year-old woman presented to our hospital with weakness and numbness in the left upper and lower limbs. She had experienced numbness in the left chest and back for six months before presentation. The patient had no history of myelitis, optic neuritis, or malignancy. Physical examination revealed left-sided hemiparesis without facial muscle involvement. Neurological examination demonstrated severe weakness (1/5 muscle strength) of the left upper and lower extremities. Hyperreflexia was noted in the extremities, and the Babinski sign was positive on the left. MRI showed T2 hyperintense lesions in the cervical spinal cord (C2-C5), with focal gadolinium enhancement at C3 (Figures 1A, 1B). Serum AQP4 antibody levels were elevated (4.9 U/mL; reference range: <3.0 U/mL, measured using enzyme-linked immunosorbent assay (ELISA)). Cerebrospinal fluid analysis revealed a cell count of 4/mm³ (reference range: <5/mm³; 100% mononuclear cells), protein concentration of 33 mg/dL (reference range: 23-38 mg/dL), and cytology class Ⅱ. Based on these findings, the patient was diagnosed with NMOSD and received methylprednisolone pulse therapy (1 g/day for three days). Following this therapy, her left hemiparesis and numbness improved, and post-treatment spinal MRI showed a reduction in T2 hyperintense lesions (Figure 1C). Oral prednisolone 10 mg/day was prescribed to prevent recurrence.

**Figure 2:**
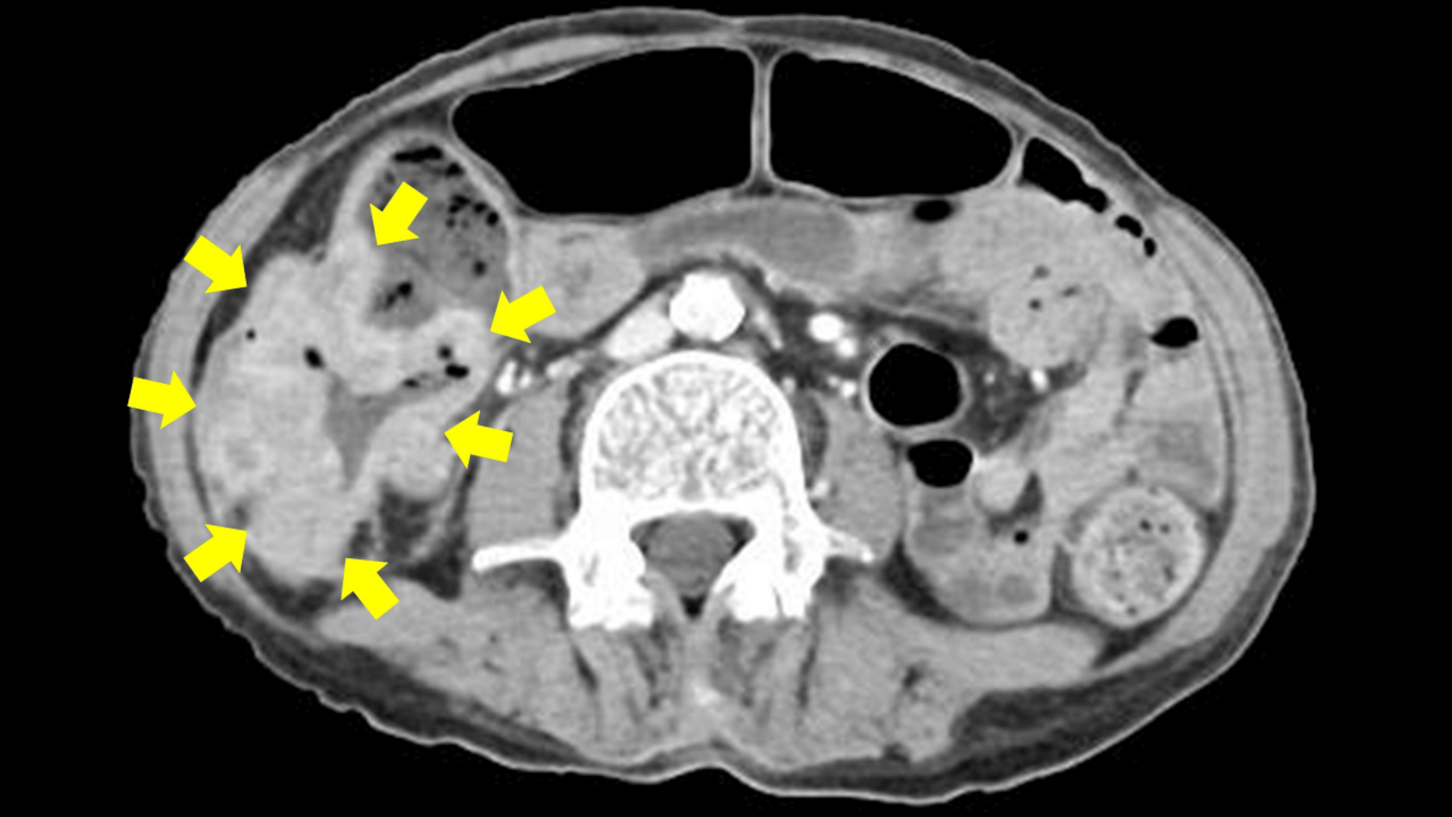
Contrast-enhanced abdominal CT demonstrating ascending colon tumor. The axial CT image shows circumferential wall thickening with irregular enhancement in a portion of the ascending colon (arrows).

**Figure 3:**
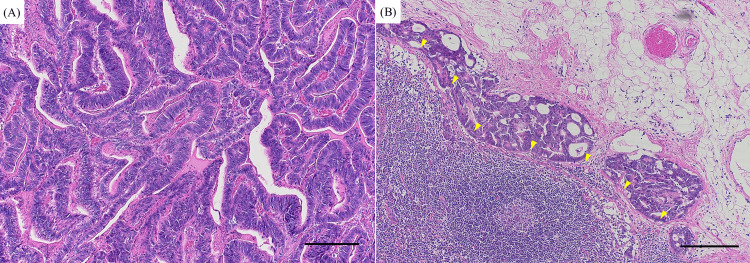
Histopathology of the primary tumor and lymph node metastasis. (A) Hematoxylin and eosin-stained section of the resected ascending colon shows adenocarcinoma. (B) Hematoxylin and eosin-stained section of an intraperitoneal lymph node demonstrates subcapsular metastatic adenocarcinoma, with arrows indicating the tumor-node interface. The scale bars represent 200 μm (A and B).

She had no abdominal symptoms, including abdominal pain, nausea, vomiting, or signs of bowel obstruction; nevertheless, anemia was detected at admission (hemoglobin: 6.4 g/dL; reference range: 11.6-14.8 g/dL), along with the presence of blood in the stool. Contrast-enhanced abdominal CT revealed a tumor in the ascending colon (Figure 2). Endoscopic evaluation could not be performed due to tumor-induced bowel obstruction. On day 44 after steroid pulse therapy, the patient underwent surgical resection of the tumor, which led to improvement in both anemia and bowel obstruction. Histopathology with hematoxylin-eosin staining of the resected specimen confirmed adenocarcinoma of the ascending colon with intraperitoneal lymph node metastasis (Figures 3A, 3B). The patient’s left hemiparesis improved, and she regained independent ambulation. She was discharged on day 53 and continued prednisolone 10 mg daily for relapse prevention. At a six-month follow-up, there was no evidence of recurrence.

**Figure 4:**
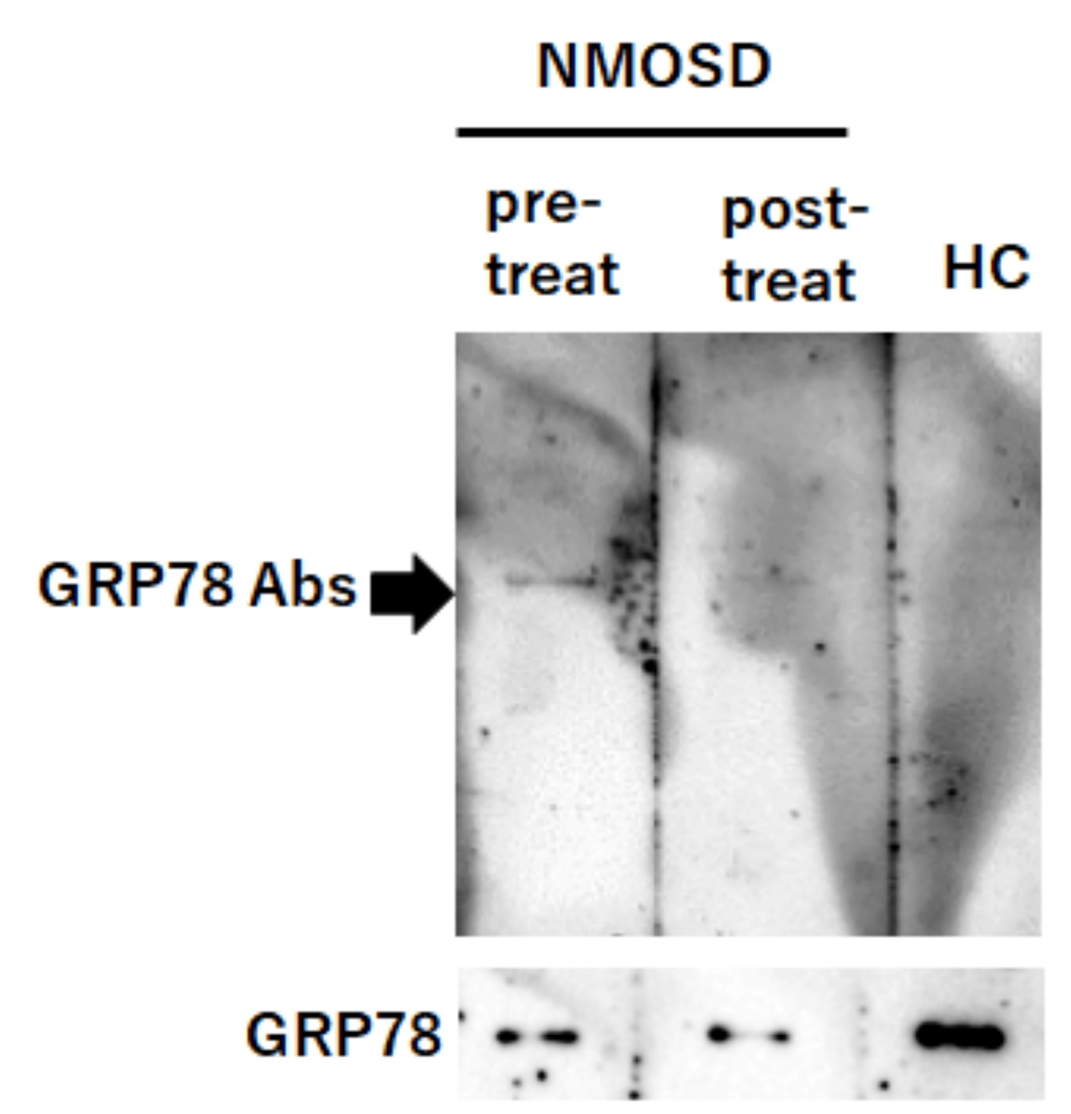
Western blot analysis for detecting GRP78 autoantibodies from the patient’s serum IgG. Recombinant full-length human GRP78 protein (1 μg; Abcam) was utilized as the antigen, separated by electrophoresis, and subsequently transferred onto a polyvinylidene difluoride membrane. IgG (5 μg/mL) from the patient’s sera pre-treatment (“pre-treat”) and post-treatment (“post-treat”) was used as primary antibodies. IgG (5 μg/mL) from an HC, and commercial GRP78 antibody (1:200 dilution, Abcam) were also utilized as primary antibodies. Detection was performed using an HRP-conjugated anti-human secondary antibody or an HRP-conjugated anti-rabbit secondary antibody (1:4,000 dilution) and was visualized by chemiluminescence (ImmunoStar® LD, Wako). A distinct band corresponding to 78-kDa GRP78 (indicated by a black arrowhead) was detected in the pre-treatment IgG sample, but was not observed in the post-treatment or HC samples. Bands were visualized by chemiluminescence using ImmunoStar® LD (Wako). Line pre-treat, band corresponding to GRP78 antibodies from patient’s sera pre-treatment; Line post-treat, band corresponding to GRP78 antibodies from patient’s sera post-treatment; Line HC, band corresponding to GRP78 antibodies from HC. Line GRP78, Rabbit anti-GRP78 antibodies were used as the positive control. NMOSD = neuromyelitis optica spectrum disorder; GRP78 = glucose-regulated protein 78; HC = healthy control; HRP = horseradish peroxidase

**Figure 5:**
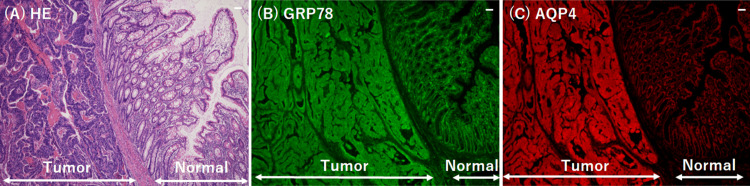
Immunohistochemical analysis of GRP78 and expression in tumor and adjacent normal colorectal tissues. (A) HE staining was used to distinguish the tumor from the surrounding non-neoplastic regions in the surgical specimen. Immunostaining of GRP78 (B) and AQP4 (C) was performed using each primary antibody (GRP78 antibody, Abcam, 1:50 dilution (B); AQP4 antibody, Abcam, 1:50 dilution (C)). Alexa Fluor 488-conjugated goat anti-rabbit IgG and Alexa Fluor 596-conjugated goat anti-mouse IgG (Invitrogen; 1:100 dilution) were utilized as secondary antibodies. GRP78 and AQP4 expression levels were elevated in tumor tissue relative to adjacent normal tissue. Scale bar: 50 μm. HE = hematoxylin and eosin; GRP78 = glucose-regulated protein 78; AQP4 = aquaporin-4

GRP78 antibodies were assayed in serum samples obtained at admission and postoperatively using methods previously described [8]. A band corresponding to GRP78 antibodies was detected in the sera collected at admission, but this became weak or at a borderline detection level in the sera collected postoperatively (Figure 4). AQP4 antibodies were also not detected after tumor resection, as assessed by ELISA. Immunohistochemical analysis of the patient’s colorectal tumor revealed higher expression of both GRP78 and AQP4 in tumor tissue compared to that in adjacent normal tissues (Figure 5).

## Discussion

A notable feature of this case was the high expression of AQP4 and GRP78 in colon cancer tissue associated with NMOSD, along with serum positivity for both AQP4 and GRP78 antibodies. Furthermore, AQP4 and GRP78 antibodies became undetectable following tumor resection, and no signs of NMOSD relapse were observed at six months postoperatively, although continued administration of prednisolone 10 mg may have contributed to disease stability.

To our knowledge, this is the first reported case of NMOSD associated with a malignant tumor showing high expression of both AQP4 and GRP78 in tumor cells, followed by the decrease of GRP78 antibodies after tumor resection. Several cases of NMOSD associated with malignancy have been documented, and AQP4 expression in tumor cells has been demonstrated in some cases [9,10]. In addition, a few reports have noted the disappearance of AQP4 antibodies after tumor resection [10,11], although patients in these reports also received acute or maintenance immunosuppressive therapies, or both. Although such treatments can reduce AQP4 antibody levels, complete seronegativity is rarely achieved [12,13]. These findings suggest that an immune response targeting tumor-associated AQP4 may lead to autoantibody production and NMOSD onset as a paraneoplastic syndrome.

In line with prior studies, AQP4 expression was confirmed in the tumor cells in this case, and anti-AQP4 antibodies became undetectable after tumor resection. GRP78 was also highly expressed in tumor tissue, and the serum was positive for GRP78 antibodies. NMOSD is caused by damage to astrocytes induced by AQP4 antibodies. Given that AQP4 antibodies cannot penetrate the central nervous system without BBB disruption, BBB breakdown is considered a critical step in NMOSD pathogenesis [14]. GRP78 antibodies have been reported in patients with NMOSD, myelin oligodendrocyte glycoprotein antibody-associated disease, paraneoplastic cerebellar degeneration with Lambert-Eaton myasthenic syndrome, and various tumors, including esophageal, gastric, and colon cancers [3,4,15,16]. GRP78 expression has also been shown to be significantly elevated in colorectal cancer tissue compared to normal tissue [11,17]. Together, these findings suggest that autoimmune targeting of tumor-expressed GRP78 may promote production of GRP78 autoantibodies in NMOSD patients with cancer [14,18]. This case demonstrates both high GRP78 expression in malignant tumor tissue and serum GRP78 antibody positivity. Following tumor resection, GRP78 antibodies became weak or were at a borderline detection level. These findings support the hypothesis that an immune response directed against tumor-expressed AQP4 and GRP78 triggered production of corresponding antibodies, with GRP78 antibodies potentially facilitating BBB disruption and contributing to paraneoplastic NMOSD development.

## Conclusions

In this case, NMOSD was associated with ascending colon cancer, and both AQP4 and GRP78 were highly expressed in the tumor tissue. Serum analysis revealed the presence of AQP4 and GRP78 antibodies. GRP78 antibodies may have contributed to the development of paraneoplastic NMOSD. Screening for and treating occult malignancies may represent a therapeutic strategy for disease management. Furthermore, GRP78 autoantibodies may play a central role in the pathogenesis of paraneoplastic NMOSD. Accumulating evidence from studies on GRP78 antibodies may help clarify the pathogenic mechanisms underlying paraneoplastic NMOSD. Further accumulation of cases is expected to clarify the role of anti-GRP78 antibodies in NMOSD.
